# Benzophenone Synthase and Chalcone Synthase Accumulate in the Mesophyll of *Hypericum perforatum* Leaves at Different Developmental Stages

**DOI:** 10.3389/fpls.2016.00921

**Published:** 2016-06-29

**Authors:** Asma K. Belkheir, Mariam Gaid, Benye Liu, Robert Hänsch, Ludger Beerhues

**Affiliations:** ^1^Institute of Pharmaceutical Biology, Technische Universität BraunschweigBraunschweig, Germany; ^2^Institute of Plant Biology, Technische Universität BraunschweigBraunschweig, Germany

**Keywords:** *Hypericum perforatum*, chalcone synthase, benzophenone synthase, polyketide synthases, immunofluorescence localization, histochemical localization, flavonoids, xanthones

## Abstract

The active medicinal constituents in *Hypericum perforatum*, used to treat depression and skin irritation, include flavonoids and xanthones. The carbon skeletons of these compounds are formed by chalcone synthase (CHS) and benzophenone synthase (BPS), respectively. Polyclonal antisera were raised against the polyketide synthases from *Hypericum androsaemum* and their IgG fractions were isolated. Immunoblotting and immunotitration were used to test the IgGs for crossreactivity and monospecificity in *H. perforatum* leaf protein extract. Immunofluorescence localization revealed that both CHS and BPS are located in the mesophyll. The maximum fluorescence levels were observed in approx. 0.5 and 1 cm long leaves, respectively. The fluorescence intensity observed for CHS significantly exceeded that for BPS. Using histochemical staining, flavonoids were detected in the mesophyll, indicating that the sites of biosynthesis and accumulation coincide. Our results help understand the biosynthesis and underlying regulation of active *H. perforatum* constituents.

## Introduction

Medications containing extracts from the flowering upper parts of the medicinal plant *Hypericum perforatum* (St. John’s wort; Hypericaceae) are used for the treatment of mild to moderate depressions as well as skin irritations and infected wounds (Linde et al., 2008; Wölfle et al., 2014). Due to the additive and synergistic effects of the ingredients, the entire extract is commonly used for therapy. The major active constituents involve hyperforins, hypericins, flavonoids, and xanthones (Beerhues, 2011). All these four classes of compounds are polyketide derivatives. Crucial steps of their biosynthetic pathways are catalyzed by polyketide synthase (PKS) enzymes. Plant PKSs (type III) are homodimers. Either subunit has an independent active site, which accommodates the starter and extender substrates (Austin and Noel, 2003). Variations in the starter molecule, the number of extender units and the mode of cyclization result in the formation of an amazing array of PKS products.

The PKSs that are involved in hyperforin, hypericin, flavonoid, and xanthone biosyntheses are isobutyrophenone, octaketide, chalcone, and benzophenone synthases, respectively (Beerhues, 2011). cDNAs encoding benzophenone synthase (BPS) and chalcone synthase (CHS) were cloned from elicitor-treated *Hypericum androsaemum* cell cultures and greenhouse-grown *H. sampsonii* plants and were functionally expressed in *Escherichia coli* (Liu et al., 2003; Huang et al., 2012). BPS and CHS catalyze decarboxylative condensations of benzoyl-CoA and 4-coumaroyl-CoA, respectively, with three molecules of malonyl-CoA. While benzoyl-CoA is also preferred by BPS from *Garcinia mangostana*, the enzyme from *Centaurium erythraea* uses 3-hydroxybenzoyl-CoA (Beerhues, 1996; Nualkaew et al., 2012). The products of the BPS and CHS reactions are benzophenones and chalcones, which are metabolized to xanthones and flavonoids, respectively (Winkel-Shirley, 2001; El-Awaad et al., 2016). Upon mutation in a single active site position, *H. androsaemum* BPS formed phenylpyrones (Klundt et al., 2009). Xanthones and flavonoids contribute to the medicinal effects of *H. perforatum* extracts. Understanding their biosynthetic pathways in *H. perforatum* requires, in addition to the knowledge of the individual biochemical reactions, information about the spatial and temporal regulation, which underlies the metabolic routes. Here, immunofluorescence localization of BPS and CHS in leaves of *H. perforatum* is reported.

The two other PKSs, isobutyrophenone and octaketide synthases, were not included in this study. No cDNA encoding isobutyrophenone synthase, the key enzyme of hyperforin biosynthesis, has so far been isolated. For octaketide synthase, cDNAs were cloned from various species, including *H. perforatum* (Abe et al., 2005; Karppinen et al., 2008; Mizuuchi et al., 2009). However, all the recombinant proteins form an incorrectly cyclized octaketide derivative. Correct cyclization leading to formation of emodin anthrone has recently been observed in elicitor-treated *Cassia bicapsularis* cell cultures (Abdel-Rahman et al., 2013). Octaketide synthase transcripts in *H. perforatum* leaves were localized by *in situ* hybridization, indicating their exclusive presence in hypericin-containing dark nodules (Karppinen et al., 2008). Therefore, octaketide synthase was not considered here.

In the present study, we focus on the localization of BPS and CHS. Antibodies were raised, tested for their specificities and used for immunofluorescence detection of the PKSs in the mesophyll of *H. perforatum* leaves. Furthermore, biosynthetic products were histochemically localized. While a specific stain for xanthones was not available, flavonoids were detected in the mesophyll.

## Materials and Methods

### Plants

*Hypericum perforatum* L. (Hypericaceae) was grown in the medicinal plants garden of the Institute of Pharmaceutical Biology, Technische Universität Braunschweig, Germany.

### Chemicals and Materials

Solvents and chemicals were of either analytical or high performance liquid chromatography (HPLC) grade. Polyvinylidene difluoride (PVDF) blotting membranes (Immobilon P) were purchased from Millipore (Bedford, USA). Enhanced chemiluminescence (ECL) Western blotting detection reagents were ordered from GE Healthcare (Freiburg, Germany). Peroxidase-conjugated AffinPure goat anti-rabbit IgG (H + L) and Alexa Fluor 488-goat anti-rabbit IgG (H + L) were obtained from Dianova (Hamburg, Germany) and Invitrogen (Karlsruhe, Germany), respectively. Cryo-embedding material and poly-L-lysine-coated slides were purchased from Plano (Marburg, Germany) and Roth (Karlsruhe, Germany), respectively. Polyclar AT and diphenylboric acid 2-aminoethyl ester (DPBA) were ordered from Serva (Heidelberg, Germany) and Sigma-Aldrich (Taufkirchen, Germany), respectively.

### Generation of Polyclonal Antisera and Purification of IgG Fractions

The BPS and CHS sequences used were from *H. androsaemum* (Liu et al., 2003). They were expressed as both His_6_-tag and GST-fusion proteins using pRSET B (Invitrogen, Karlsruhe, Germany) and pGEX (Görlach and Schmid, 1996) expression vectors, respectively. The proteins were purified by affinity chromatography using Ni–NTA agarose and GSTrap matrices, respectively (Liu et al., 2003, 2007). The His_6_-tag proteins were used for immunization of rabbits, which was carried out by SEQLAB Sequence Laboratories (Göttingen, Germany). The IgG fractions of the antisera and the pre-immune sera were isolated and stored, as described previously (Chizzali et al., 2012).

### Protein Extraction and Immunoblotting

Fresh leaves (1 g) of varying size (0.3, 0.5, 0.8, 1.5, and 2.0 cm) were frozen in liquid nitrogen, ground in a mortar, mixed with 10% (w/v) Polyclar AT and extracted on ice for 10 min with 1 ml 50 mM Tris–HCl pH 7.4 containing 10 mM 1,4-dithiothreitol (DTT), 0.5 mM sucrose and 1 mM phenylmethane sulphonyl fluoride (protease inhibitor). The homogenate was centrifuged at 8,900 g for 25 min and the supernatant was used for immunoblotting. Protein concentration was determined by the method of Bradford (1976). Soluble proteins were separated on a 12% (w/v) sodium dodecyl sulphate (SDS) polyacrylamide gel and electroblotted on a PVDF membrane, as described previously (Chizzali et al., 2012). After blocking, the membrane was incubated with anti-His_6_–BPS IgG (1:100,000 v/v) and anti-His_6_–CHS IgG (1:10,000 v/v). Incubation with peroxidase-conjugated goat anti-rabbit IgG and further processing were carried out, as described previously (Chizzali et al., 2012). As control for efficient blotting, the membrane and the gel were stained with Indian ink and Coomassie blue solutions, respectively.

### Enzyme Assays

The incubation mixtures (250 μL) consisted of 0.1 M potassium phosphate pH 7.0, 324 μM malonyl-CoA and 2 μg protein. In addition, the BPS and CHS assays contained 54 μM benzoyl-CoA and 4-coumaroyl-CoA, respectively. After incubation at 30°C for 10 min, the enzymatic products were extracted and analyzed by high performance liquid chromatography (HPLC), as described previously (Liu et al., 2003).

### Immunotitration

Mixtures of enzyme solution (50 μl) and IgG solution (50 μl of 1:2 to 1:512 dilutions) were incubated for 20 min at room temperature (Beerhues and Wiermann, 1988). Phosphate-buffered saline (PBS) (50 μl) containing 6% (w/v) polyethylene glycol 8000 was added. Following incubation at 4°C over night, the mixtures were centrifuged for 10 min at 8,700*g*. An aliquot of the supernatant (100 μl) was used to determine the non-precipitated enzyme activity that remained in the supernatant. Controls without antibody and with 1:2 to 1:512 dilutions of the pre-immune IgG were included.

### Immunofluorescence Localization of BPS and CHS

For tissue fixation, the method of Moll et al. (2002) was used, except for slight modifications adapted to *H. perforatum* tissue. Small segments (1.2 mm^2^) were immediately fixed for 2 h under reduced pressure (0.3 mbar) in ice-cold buffered fixative solution, which consisted of 2% w/v formaldehyde (freshly prepared from paraformaldehyde), 0.1% v/v glutaraldehyde and 0.1% v/v Triton X-100 in 0.1 M phosphate buffer pH 7.2. After washing with PBS (2 × 10 min), the samples were dehydrated in a graded ethanol series (30, 50, 70, and 90% for 30 min each at room temperature). For cryosectioning, fixed and PBS-washed tissue was embedded in a cryo-embedding matrix and stored in a cool and dry place. The specimens were cut to thin segments (18–20 μm) using a cryomicrotome (HM 500 O cryostate, Microme). The sections were transferred to poly-L-lysine-coated slides, dried and further treated, as described previously (Chizzali et al., 2012). The pre-immune IgG and His_6_-tag IgG fractions were used in 1:10 to 1:100 dilutions. Goat anti-rabbit secondary antibody was conjugated with Alexa Fluor 488, which exhibits absorbance of blue light at 494 nm and emission of green light at 517 nm.

### Histochemical Localization of Flavonoids

Fresh hand-sectioned leaves were stained for 5 min with 0.125% (w/v) diphenylboric acid 2-aminoethyl ester (DPBA) in 0.005% (v/v) Triton X-100 and washed in water for 2 min. Images were taken using the confocal laser scanning microscope cLSM-510META (Release Version 4.2 SP1), connected to an Axiovert 200M (Carl Zeiss). The specimens were examined either using the Plan-Neofluar 10×/0.3 for overview or the C-Apochromat 40×/1.2 water-immersion objective for detailed pictures. The settings were as follows. Flavonoid staining was recorded using the 488 nm argon-laser (14% intensity) and chlorophyll autofluorescence was recorded using the 633 nm Helium laser (45% intensity) for excitation in the multi-tracking mode. The emitted light passed the primary beam splitting mirrors UV/488/543/633 and was detected after splitting with the NFT-545 on BP 505–550 for flavonoid staining and LP 650 for chlorophyll detection, respectively. When appropriate, the bright-field images of samples were visualized using the transmitted light photomultiplier. The lambda-mode was used to examine the spectral signature of fluorophores. All images were processed using the LSM Image Browser Release 4.2 (Carl Zeiss).

## Results

### Antisera and Isolated IgG Fractions

**FIGURE 1:**
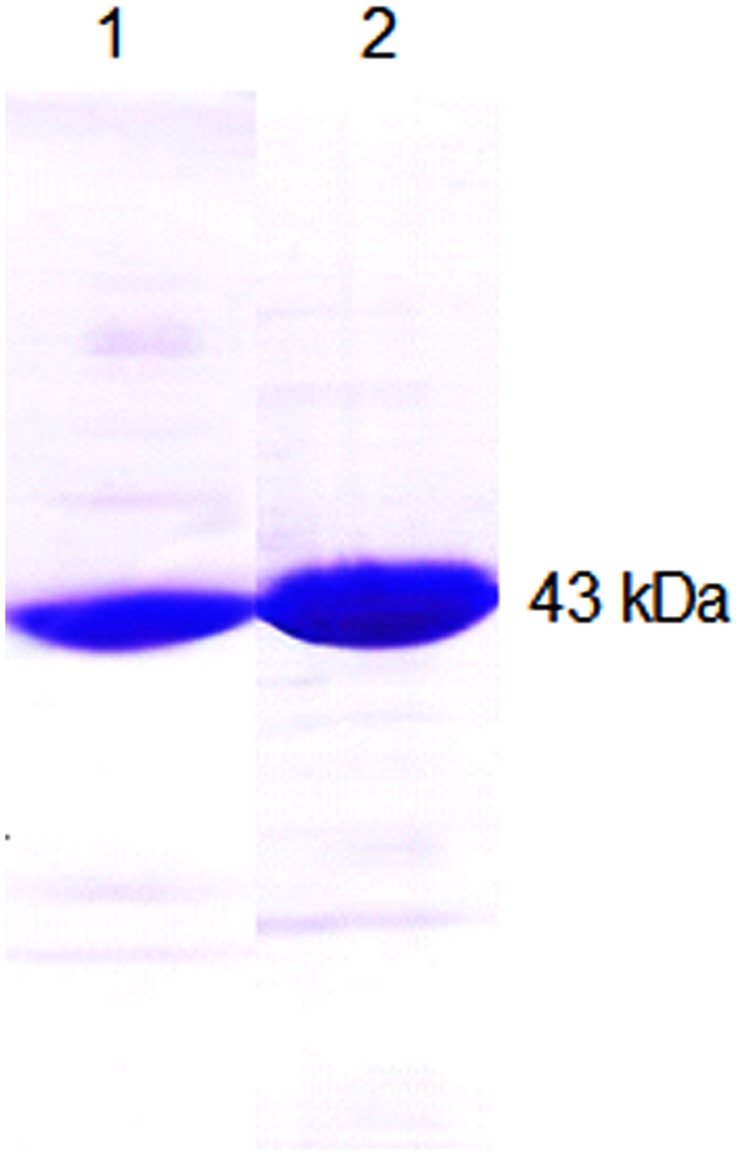
**Overexpression and affinity chromatography yielded proteins for immunization of rabbits.** Purified His_6_– benzophenone synthase (BPS) **(1)** and His_6_– chalcone synthase (CHS) **(2)** were run on a sodium dodecyl sulphate (SDS) polyacrylamide gel, indicating near-homogeneity each.

To raise antibodies against BPS and CHS, the coding sequences of the proteins from *H. androsaemum* (Liu et al., 2003) were expressed in *E. coli* to yield both His_6_-tag and GST-fusion proteins. Affinity chromatography on Ni–NTA and GSTrap matrices, respectively, resulted in proteins of near-homogeneity each, as indicated by SDS–polyacrylamide gel electrophoresis (PAGE) (**Figure 1**). The His_6_-tag proteins served to raise polyclonal antisera in rabbits and the IgG fractions were isolated from both the pre-immune sera and the antisera. When studied by SDS–PAGE, the heavy and light chains at 50 and 25 kDa, respectively, were the only bands detectable. The isolated IgG fractions were tested for crossreactivity and monospecificity.

### Lack of Crossreactivity between BPS and CHS Antibodies

Parallel immunolocalization of BPS and CHS requires that the antibodies do not crossreact with the respectively other antigen. Immunoblotting and immunotitration were used to study the specificities of the IgG fractions isolated. To rule out that cross-reactions in the polyclonal antisera occur between the His_6_ tag and His_6_-tag-directed antibodies, the GST-fusion proteins were used as antigens.

**FIGURE 2:**
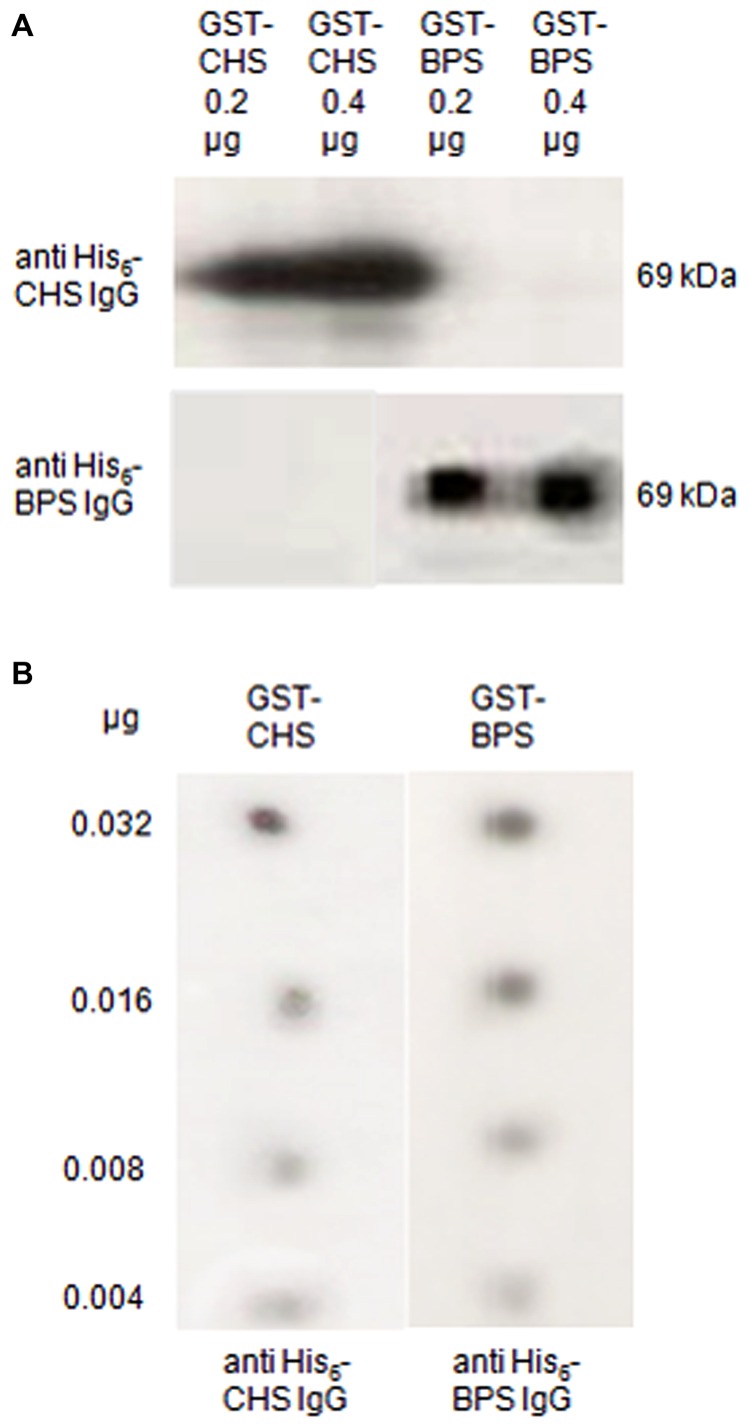
**The BPS and CHS antibodies lacked crossreactivity and resembled in detection sensitivity.** **(A)** Immunoblotting of CHS and BPS. The GST-fusion proteins were run on a SDS polyacrylamide gel, blotted on a polyvinylidene difluoride (PVDF) membrane and incubated with the antibodies raised against the His_6_-tag proteins. **(B)** Dot blotting. Decreasing quantities of the GST-fusion proteins were dotted on a PVDF membrane and immunostained with anti-His_6_–CHS IgG and anti-His_6_–BPS IgG.

In immunoblotting after SDS–PAGE of the affinity-purified GST-fusion proteins, anti-His_6_–CHS detected GST–CHS (69 kDa) but anti-His_6_–BPS did not (**Figure 2A**). Conversely, anti-His_6_–BPS stained GST–BPS (69 kDa) but anti-His_6_–CHS did not. Thus, no crossreactivity was observed in immunoblotting. The pre-immune IgG fractions failed to cause any immunoreactions.

Parallel immunolocalization also requires that the two IgG fractions have similar detection capacities. For dot blotting, decreasing quantities of the GST-fusion proteins were dotted on a membrane and immunostained. Both anti-His_6_–BPS and anti-His_6_–CHS detected their antigens down to 0.004 μg, indicating similar sensitivities (**Figure 2B**).

**FIGURE 3:**
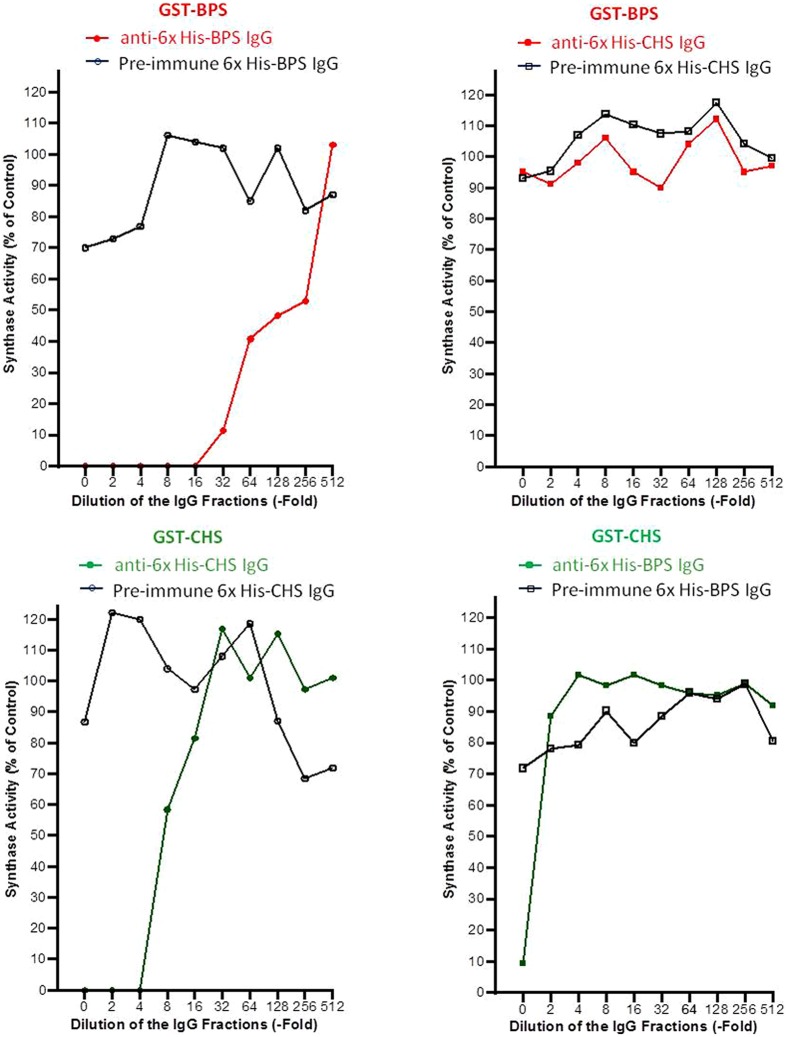
**The specific recognition of the antigens was confirmed by immunotitration.** GST–BPS and GST–CHS were used as antigens in constant quantities (2 μg). Decreasing amounts of the anti-His_6_ and pre-immune IgG fractions (1:2–1:512 dilutions) were added. The BPS and CHS activities that were not precipitated and remained in the supernatants of the titration mixtures were determined.

For immunotitration coupled with the determination of enzyme activity, first the stability of the PKSs was studied. BPS and CHS lost approx. 20 and 55%, respectively, of their activities within a day, however, the residual activities were sufficient for carrying out immunotitration. Constant quantities of the GST fusion proteins (2 μg) were mixed with decreasing quantities (1:2 to 1:512 dilutions) of the pre-immune and His_6_–IgG fractions. The PKS activities that remained in the supernatants of the titration mixtures were determined (**Figure 3**). Anti-His_6_–CHS IgG precipitated GST–CHS and did not crossreact with GST–BPS. Anti-His_6_–BPS IgG precipitated GST–BPS and exhibited, when undiluted, crossreactivity with GST–CHS. However, the undiluted IgG fractions were not used for immunolocalization. Pre-immune His_6_–CHS IgG did not recognize the PKSs, whereas pre-immune His_6_–BPS IgG resulted in weak precipitation of the proteins.

### Antibody Monospecificity in Leaf Extract

**FIGURE 4:**
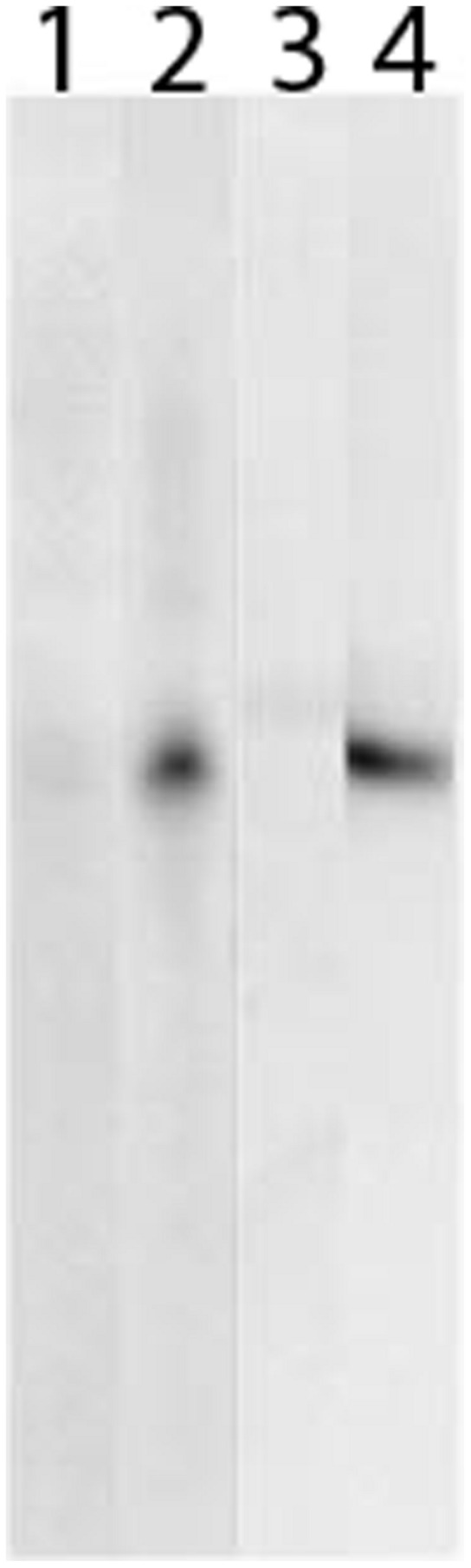
**Both BPS and CHS antibodies reacted monospecifically in leaf protein extract.** Leaf proteins were separated by SDS–PAGE, blotted on a PVDF membrane and incubated with BPS pre-immune IgG **(1)**, anti-His_6_–BPS IgG **(2)**, CHS pre-immune IgG **(3)** and anti-His_6_–CHS IgG **(4)**.

For use in immunolocalization, the BPS and CHS antibodies must (i) not crossreact with each other and (ii) not crossreact with foreign proteins, which occur in the leaf. Therefore, protein extracts from *H. perforatum* leaves at different developmental stages were subjected to SDS–PAGE and subsequent immunoblotting (**Figure 4**). Both anti-His_6_–BPS IgG and anti-His_6_–CHS IgG detected a single protein band at approx. 43 kDa, which corresponds to the subunit molecular mass of BPS and CHS. Thus, monospecificity in leaf protein extract was demonstrated for both IgG fractions. No staining of protein bands was observed when the two pre-immue IgG fractions were used.

### Immunolocalization of BPS and CHS to the Mesophyll

Localization by immunofluorescence was carried out with leaves of field-grown plants, using green fluorescent labeling (Alexa Fluor 488) and laser scanning confocal microscopy. Two alternative procedures of section preparation were examined, the resin (Technovit) and the cryo-sectioning techniques. Since resin-embedded sections exhibited strongly decreased PKS antigenicities and strong unspecific background labeling, cryo-sectioning was preferred. Best results, i.e., high level of specific detection and low level of background staining, were obtained with 1:25 dilutions of the IgG fractions. In addition, this dilution failed to cause any crossreactivity between the BPS and CHS IgG preparations, as described above.

**FIGURE 5:**
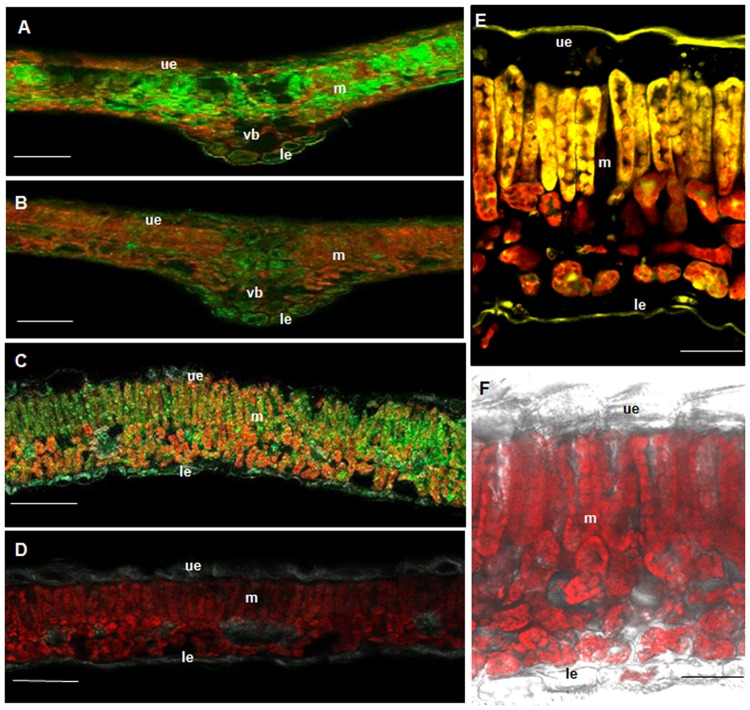
**BPS and CHS as well as flavonoids are located in the mesophyll.** Immunofluorescence localization of CHS **(A,B)** and BPS **(C,D)** using a green fluorescent dye. **(A,C)** Cross-sections incubated with anti-His_6_–CHS IgG and anti-His_6_–BPS IgG, respectively. **(B,D)** Cross-sections incubated with the pre-immune IgGs. Localization of flavonoids **(E,F)** using histochemical detection. **(E)** Staining of flavonoids with diphenylboric acid 2-aminoethyl ester (DPBA). **(F)** Control section exhibiting only the red autofluorescence of chlorophyll. ue, upper epidermis; le, lower epidermis; m, mesophyll; vb, vascular bundle. Bar, 100 μm **(A–D)**, 20 μm **(E,F)**.

For CHS, bright immunofluorescence was observed in the mesophyll (**Figure 5A**). Palisade and sponge cells exhibited similar staining intensities. The lambda signature of the Alexa Fluor 488-labeled sections verified the correct emission wavelength (520 nm). Epidermal tissue was devoid of immunostaining. No labeling was observed in control sections incubated with pre-immune IgG (**Figure 5B**).

BPS was also present in the mesophyll, however, the intensity of immunofluorescence was markedly lower than for CHS (**Figure 5C**). The upper and the lower epidermis were devoid of staining. No labeling was observed with pre-immune IgG (**Figure 5D**).

### Histochemical Localization of Flavonoids to the Mesophyll

To stain the CHS products in *H. perforatum* leaf cross-sections, diphenylboric acid 2-aminoethyl ester (DPBA) was used (**Figure 5E**). Flavonoids were present in the mesophyll, palisade cells exhibiting stronger staining than sponge cells. The epidermal layers were devoid of labeling. No staining was observed in control sections (**Figure 5F**). For histochemical localization of xanthones, no specific stain was available.

### Distinct Developmental Regulation of BPS and CHS

**FIGURE 6:**
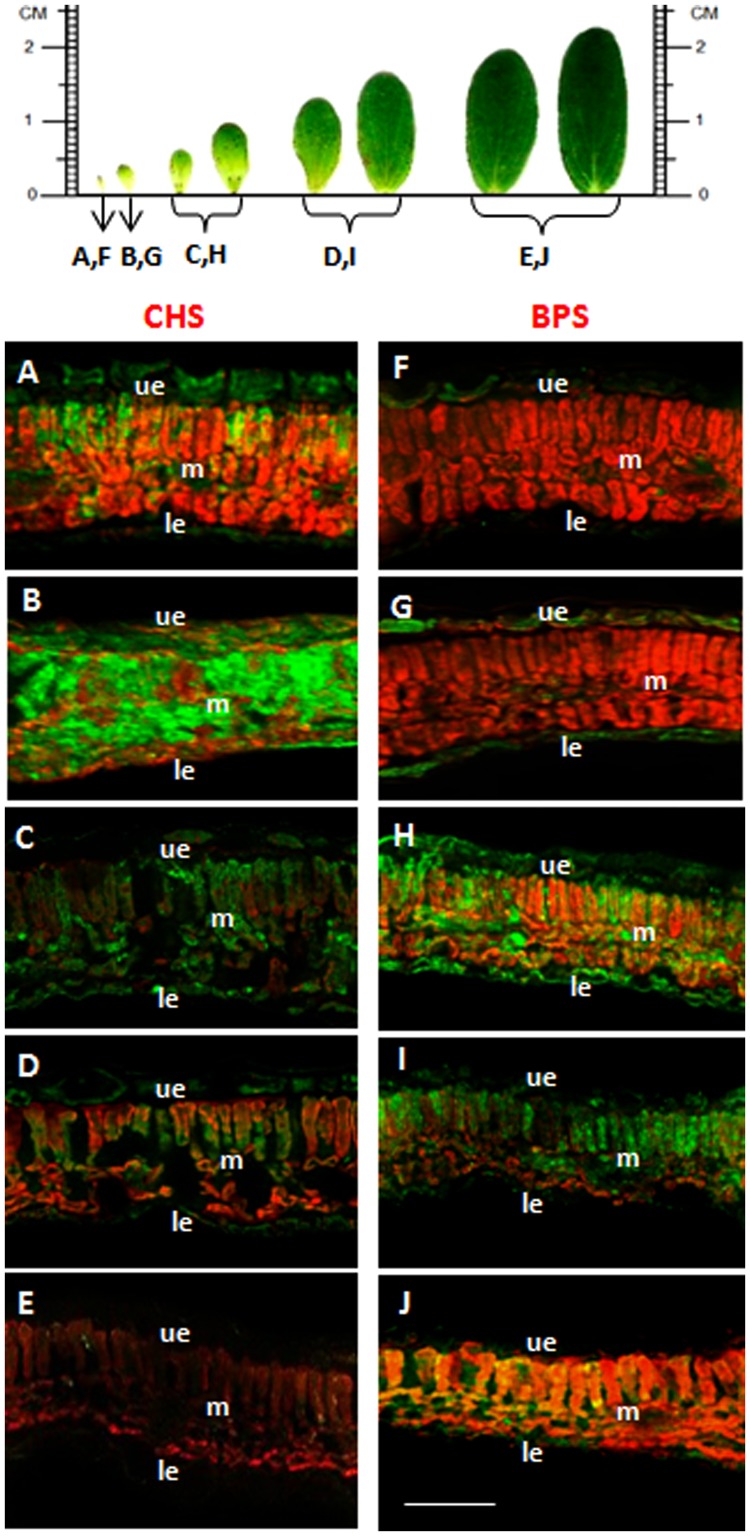
**The developmental stages of maximum BPS and CHS levels differ.** CHS **(A–E)** and BPS **(F–J)** were immunolocalized using a green fluorescent dye. Sections were prepared from leaves with a blade length of approximately 0.3 cm **(A,F)**, 0.5 cm **(B,G)**, 0.8 cm **(C,H)**, 1.5 cm **(D,I)** and 2.0 cm **(E,J)**. ue, upper epidermis; le, lower epidermis; m, mesophyll. Bar, 100 μm.

Leaves at various developmental stages were cross-sectioned and incubated with anti-His_6_–BPS IgG and anti–His_6_-CHS IgG in different sets of experiments. The intensity of immunofluorescence in the mesophyll changed with leaf age, which held true for both CHS and BPS (**Figure 6**). Maximum immunolabeling of CHS was observed in approx. 0.5 cm long leaves, which lacked detectable BPS quantities. The CHS-specific fluorescence rapidly decreased to a basal level in approx. 1 cm long leaves which, however, exhibited a high level of BPS immunofluorescence. In elder leaves, BPS fluorescence declined.

## Discussion

In the medicinal plant *H. perforatum*, the major active metabolites are formed by polyketide synthases, two of which are BPS and CHS. Demonstrated herein is that both BPS and CHS are located in *H. perforatum* leaves in the mesophyll tissue. Given the comparable detection capacities of the antibodies used, the immunofluorescence intensities for BPS and CHS differed significantly. The CHS level markedly exceeded the BPS level, which is in accordance with the previously detected quantities of flavonoids as CHS products and xanthones as BPS products. The flavonoid content in the aerial parts was 2–4%, the major compounds being quercetin derivatives, such as hyperoside and rutin (Nahrstedt and Butterweck, 1997; Hölzl and Petersen, 2003). In contrast, only traces of xanthones, such as 1,3,6,7-tetrahydroxyxanthone and mangiferin, were detected. As an exception, the aerial parts of Indian *H. perforatum* contained 2–4% xanthones (Muruganandam et al., 2000). Commonly, xanthones are abundant in roots of *Hypericum* species, which is consistent with the high BPS transcript level found in *H. sampsonii* roots (Pasqua et al., 2003; Huang et al., 2012; Zubrická et al., 2015). Therefore, immunohistochemical studies of these organs will be interesting.

CHS and BPS accumulated at different stages of leaf development, with CHS accumulation occurring earlier than that of BPS. Consistently, relatively high BPS transcript levels were detected in older leaves of *H. sampsonii*, whereas younger leaves had relatively high CHS transcript levels (Huang et al., 2012). Expression of *CHS* in young leaves is physiologically explainable. Due to absorption at 280–315 nm, flavonoids efficiently protect photosynthetically active tissue from damaging UV-B radiation, which can penetrate the ozone layer in the stratosphere (Harborne and Williams, 2000). Flavonoids also function as a preformed barrier against herbivore attack (Winkel-Shirley, 2001). As a consequence, flavonoid accumulation has to be initiated at an early stage of leaf development. In contrast, xanthones in *Hypericum* species serve as inducible defense compounds against microbial pathogens, i.e., phytoalexins (Abd El-Mawla et al., 2001; Franklin et al., 2009). Cell cultures of *H. perforatum* accumulated xanthones in response to the addition of a fungal elicitor prepared from *Colletotrichum gloeosporioides*, the causal agent of St. John’s wort wilt (Gärber and Schenk, 2003; Conceição et al., 2006). Furthermore, *H. androsaemum* and *H. calycinum* cell cultures accumulated prenylated xanthones upon challenge with elicitors and a cDNA encoding the prenyltransferase involved was isolated (Abd El-Mawla et al., 2001; Gaid et al., 2012; Fiesel et al., 2015).

In *H. perforatum* leaves, the CHS products were located in the mesophyll tissue, as indicated by histochemical staining. Thus, the sites of biosynthesis and storage of flavonoids are identical. No transport process takes place at the tissue level. Previously, a similar tissue distribution of flavonoid biosynthetic enzymes was observed in primary leaves of oat (*Avena sativa*) by peeling the epidermal layers (Knogge and Weissenböck, 1986). The entire pathway, including CHS, chalcone–flavanone isomerase and methyl- and glycosyltransferase activities, was located in the leaf mesophyll. However, flavonoids, in this case flavones, were found in both epidermis and mesophyll tissues, with up to 70% being detected in the two epidermal layers (Weissenböck and Sachs, 1977). Therefore, intercellular translocation of individual products was proposed. While vitexin derivatives are transported to the epidermis, isovitexin derivatives remain in the mesophyll. Alternatively, the epidermal product pattern may reflect flavonoid biosynthesis in the subepidermal mesophyll cells.

Strict compartmentation of flavonoids between tissues and a close correlation between leaf development and flavonoid metabolism were also observed in primary leaves of rye (*Secale cereale*; Schulz et al., 1985; Schulz and Weissenböck, 1986; Hutzler et al., 1998). While two *C*-glucosylapigenin-*O*-glycosides were accumulated in the two epidermal layers, two anthocyanins and two luteolin *O*-glucuronides were exclusively located in the mesophyll, as shown by isolation and separation of epidermal and mesophyll protoplasts. Maximum product accumulation coincided with maximum activities of selected flavonoid biosynthetic enzymes, such as glucuronosyltransferases (Schulz and Weissenböck, 1988).

A different tissue distribution of CHS and flavonoids than in primary leaves of grass seedlings was found in leaves of spinach (*Spinacia oleracea*), pea (*Pisum sativum*), and bean (*Vicia faba*), using immunofluorescence localization (Beerhues and Wiermann, 1988; Beerhues et al., 1988). CHS was present in the upper and the lower epidermis and to a minor extent in the subepidermal layers at an early developmental stage. CHS in leaves of spinach, pea and bean was restricted to the epidermal tissue and flavonoids were either exclusively or predominantly present in the epidermal layers, indicating that the sites of biosynthesis and storage were identical (Tissut and Ravanel, 1980; Hrazdina et al., 1982; Weissenböck et al., 1984, 1986). This was also true for parsley (*Petroselinum crispum*) leaves (Jahnen and Hahlbrock, 1988; Schmelzer et al., 1988). Using *in situ* hybridization, immunohistochemistry, and microspectrophotometry, light-induced CHS mRNA, CHS protein, and flavonoid products, respectively, were localized to epidermal cells, which thus contained the entire sequence of product formation. In leaves of *Catharanthus roseus*, CHS transripts and flavonoids were also co-localized by *in situ* hybridization and histochemistry to the epidermis, mainly the adaxial layer (Mahroug et al., 2006). Furthermore, epidermal tissue of needles of Scots pine (*Pinus sylvestris*) contained both CHS mRNA and products (Schnitzler et al., 1996).

For localization of xanthones, no specific staining was available and, even if, the low xanthone level was likely to be below the detection limit. *In vitro* regenerated shoots of *H. perforatum* even lacked detectable quantities of xanthones (Pasqua et al., 2003). An interesting alternative for xanthone localization may be leaves of Indian *H. perforatum*, which contain a high level of 1,3,5-trihydroxyxanthone derivatives (Muruganandam et al., 2000).

## Author Contributions

AB, MG, BL, RH, and LB designed the research and analyzed data; AB carried out the immunochemical studies; MG performed the histochemical analysis; MG and LB prepared the manuscript.

## Conflict of Interest Statement

The authors declare that the research was conducted in the absence of any commercial or financial relationships that could be construed as a potential conflict of interest.
